# Theranostics Applications of Nanoparticles in Cancer Immunotherapy

**DOI:** 10.3390/medsci6040100

**Published:** 2018-11-09

**Authors:** Yihai Liu, Xixi Wang, Mubashir Hussain, Mu Lv, Xiaohan Dong, Tianying Wang, Xueqin Xu, Bin Liu

**Affiliations:** 1 Department of Biomedical Engineering, School of Biomedical Engineering and Informatics, Nanjing Medical University, Nanjing 211166, China; lyh1204913205@gmail.com (Y.L); wxxwq666@163.com (X.W.); 15651738713@163.com (M.L.); xiaohantung@163.com (X.D.); wty0118@icloud.com (T.W.); jiuajiu9@outlook.com (X.X.); 2 State Key Laboratory of Bioelectronics, School of Biological Science and Medical Engineering, Southeast University, Nanjing 210096, China; mubashir_tuf@hotmail.com; 3 Key Laboratory of Clinical Medical Engineering, Nanjing Medical University, Nanjing 211166, China

**Keywords:** nanoparticles, cancer, immunotherapy, immune evasion

## Abstract

With the advancement in the mechanism of immune surveillance and immune evasion in cancer cells, cancer immunotherapy shows promising results for treating cancer with established efficacy and less toxicity. As a result of the off-target effect, the approach for delivering vaccines, adjuvants, or antibodies directly to tumor sites is gaining widespread attention. An effective alternative is to utilize nanoengineered particles, functioning as drug-delivery systems or as antigens themselves. This article reviews the practical implementation of nanotechnology in cancer immunotherapy.

## 1. Introduction

In the past decades, a number of therapeutic approaches were available for cancer treatment, including surgery, radiation, and other strategies, some of which have been awarded previous Nobel Prizes [1,2,3]. However, this year, the 2018 Nobel Prize in Physiology or Medicine was award jointly to James P. Allison and Tasuku Honjo for their discovery of cancer therapy by inhibition of negative immune regulation. Cancer immunotherapy is a novel and promising approach for treating cancers and was regarded as the breakthrough of 2013 by Science [4]. Tumor cells could evade immune surveillance, one of the core hallmarks of cancer, which laid the foundations for cancer immunotherapy [5]. Specifically, cancer vaccines or adjuvants can potentiate cytotoxic lymphocytes and activate antigen presenting cells, such as macrophages, dendritic cells, and so on, to fight cancers. However, the low targeting effect and anti-cancer efficient limited the application of cancer immunotherapy.

Nanotechnology provides a new approach for providing strengthening in targeting effect and controlled release of drugs, where researchers have produced nanoscale materials with unique optical, physical, and electrical properties to encapsulate drugs and to deliver therapeutic agents to sites of interest. Nanoparticles (NPs) protect the cargo from degradation, prolonging the circulation time and promoting local concentration in tumors as a result of their abnormal vascular architecture and enhanced permeability and retention (EPR) effects [6]. In immune aspects, nanoparticles are utilized as either delivery systems to enhance antigen processing and/or as immunostimulant adjuvants to activate or enhance immunity [7]. Furthermore, it raises the extensive interest of studies that nanoparticles contribute to the treatment of metastasis by inhibiting endothelial-to-mesenchymal transition and killing circulating tumor cells [8]. For example, Bevacizumab with CRLX101, an investigational nanoparticle-drug conjugate, showed a complementary efficacy in the treatment of metastatic triple-negative breast cancer [9].

Overall, the advancement in nanoparticle-based delivery system enhances the development of nanoimmunotherapy by combinative knowledge of the tumor microenvironment and anti-tumor immunity.

## 2. The Targets of Nanoimmunotherapy

There are two types of immune response, namely innate immunity, mediated by phagocytes and dendritic cells, and adaptive immunity, mediated by T cells and B cells.

It is known that neutrophils are important effectors of the antigen-dependent cell-mediated cytotoxicity effect, a strategy of hijacking neutrophils is designed to increase therapeutic NP deposition in tumor sites. Researchers confirmed that albumin NPs are capable of in situ lifting neutrophils with the help of a monoclonal antibody TA99 [10].

Phagocytes are shaped like a double-edged sword, which can swallow both foreign antigens and nanoparticles, and the latter will decrease the biological concentration in circulation. However, Luo et al. reported a vaccine based on a synthetic polymeric nanoparticle that functions as an immunogenic adjuvant to type 1 interferon-stimulated gene, turning phagocytes from enemies to allies against cancer [11].

Dendritic cells (DCs) play a key role in activating adaptive immune responses, so nanoparticles targeting DCs may be beneficial. A vaccine targeting NY-ESO-1 to the dendritic cell receptor DEC-205 elicits robust antigen-specific immune responses in preclinical models [12].

Generally, it is a good idea to employ nanoparticles to deliver cytokines to activate T cells. Researchers have engineered antigen-capturing nanoparticles (AC-NPs) to improve the efficacy of cancer immunotherapy significantly, which induced an expansion of CD8+ cytotoxic T cells and increased both CD4+T/regulatory T cell (Treg) and CD8+T/Treg ratios [13]. T cell transplantation is a promising method to treat immunodeficiency states owing to the cytokines produced by tumor cells. However, it remains difficult to trace the physiologic interaction between T-cells and tumor cells. A report indicates that labelled T cells with gold nanoparticles as a contrast agent allows examination of the distribution, migration, and kinetics of T-cells [14]. 

### 2.1. Targeting Immune Mediators

**Figure 1:**
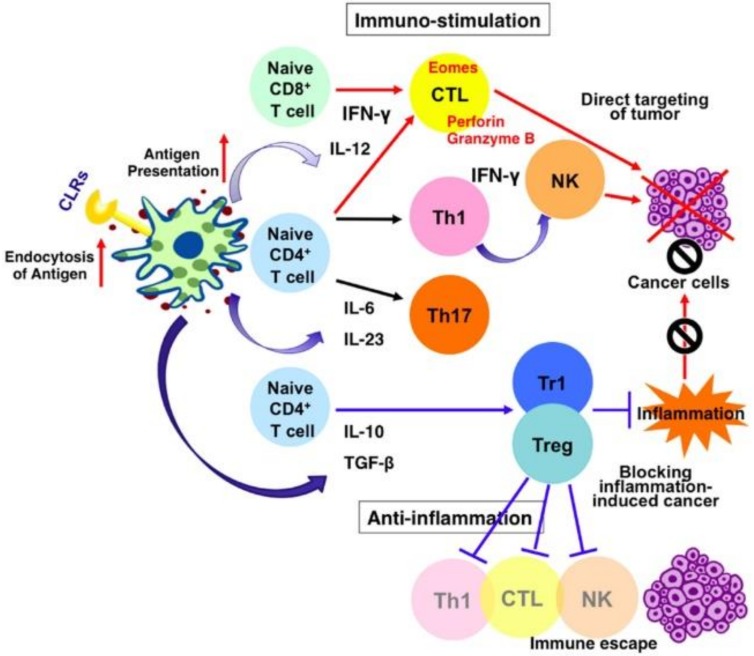
Targeting immune mediators [18]. CLR—C-type lectin receptors; CTL—cytotoxic T lymphocyte; IFN—interferons; IL—interleukins; NK—natural killer; TGF-β—transforming growth factor β; Th—T helper cell; Treg—regulatory T cell.

In addition to immune cells, the major modulators of cancer progression, cytokines are also the key targets of cancer immunotherapy. On one hand, cytokines such as interferons (IFN-γ, IFN-α) and interleukins (IL-2, IL-12) show anti-tumor capacity. On the other hand, several proinflammatory cytokines (IL-1, IL-6, Tumor necrosis factor-α (TNFα)) and Transforming growth factor-β (TGF-β) are the major immune suppressive cytokines, as shown in Figure 1.

The fundamental mechanism indicates that either suppressing the local concentration of pro-tumor cytokines, chemokines, and growth factors, or enhancing of anti-tumor immune mediators will help to improve the efficiency of cancer immunotherapy. Systemic delivery of liposomal IL-2 and TNF-α have significantly reduced the tumor growth [15,16]. Marrache et al. reported that mitochondria-targeted nanoparticles based on biodegradable polymer and zinc phthalocyanine photosensitizer can activate DCs to produce high levels of IFN-γ for cancer immunotherapy, mediated by an IL-12/IL-18 autocrine effect [17].

### 2.2. Targeting Tumor Micro-Environment

**Figure 2:**
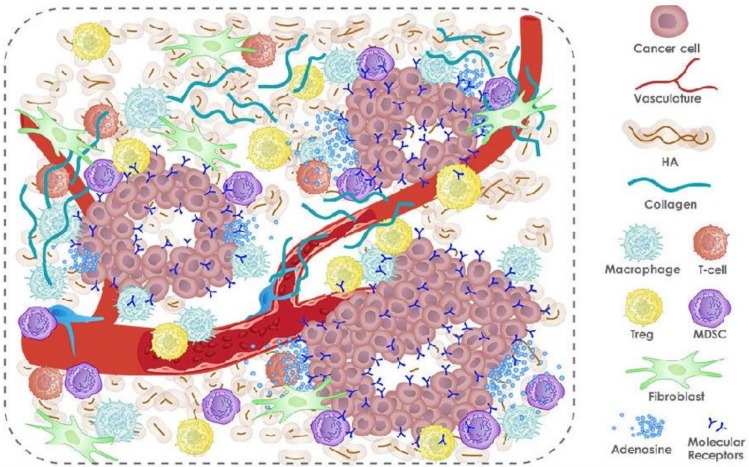
Tumor microenvironment. HA—hyaluronic acid; MDSC—myeloid-derived suppressive cell.

Tumor vascular penetration and lymphatic dysfunction allow for EPR, which forms the foundation of passive tumor targeting of NPs. However, the abnormal tumor microenvironment limits the transport of NPs deep into tumors by forming several delivery barriers, including abnormal vasculatures, dense collagen matrix, elevated interstitial fluid pressure, and collapsed vessels [19]. By normalizing the tumor microenvironment, as shown in Figure 2, drugs loaded with anti-angiogenesis, anti-fibrotic, and pH-modulating are likely to effectively kill cancer cells.

In the tumor microenvironment, decreasing immune response is attributed to tumor-associated macrophages, myeloid-derived suppressor cells, tumor-associated neutrophils, and regulatory T cells. A number of immunomodulatory and immunostimulatory molecules such as cytokines, chemokines, and targeted antibodies have been identified for their important roles in countering the highly immunosuppressive tumor microenvironment, where nanoparticles can point out these immunosuppressive cells by targeting the spleen and lymph.

### 2.3. Targeting Immune Checkpoints

**Figure 3:**
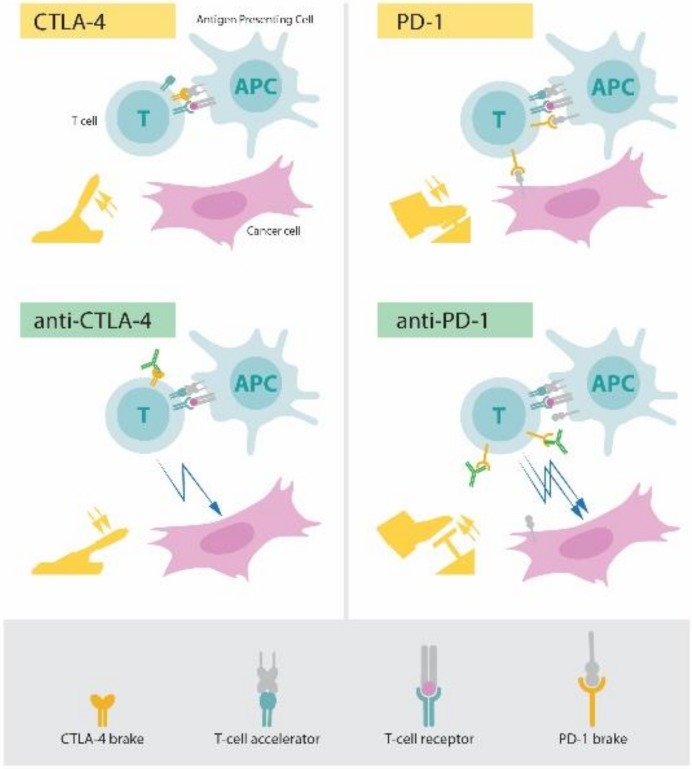
The principle for immune therapy. APC—antigen presenting cell; CTLA-4—cytotoxic T-lymphocyte antigen 4; PD-1—programmed death 1.

There are some molecules that play a key role in checkpoint regulation, and the most widely investigated are T-lymphocyte antigen 4 (CTL4) and the programmed death-1 (PD1) protein, as shown in Figure 3. Ipilimumab (anti-CTLA-4) and pembrolizumab (anti-PD-1) are approved by the US Food and Drug Administration for the treatment of advanced melanoma [20]. The checkpoint blockade with mAbs is effective in inhibiting pathways that keep the duration and strength of the immune system in check.

Prof. Gu constructed a microneedle patch, composed of biocompatible hyaluronic acid integrated with pH-sensitive dextran nanoparticles that encapsulate PD1, inducing robust immune responses in a B16F10 mouse melanoma model compared with MN without degradation or injection of free artificial PD1 [21]. They also construct an inflammation-triggered combination delivery of anti-PD-1 antibody and CpG oligodeoxynucleotides (CpG ODNs) and demonstrated that these nanoparticles can prevent cancer relapse utilizing postsurgical inflammatory response [22]. On the other hand, they developed a synergistic immunotherapy strategy that locally targets the immunoinhibitory receptor PD1 and immunosuppressive enzyme indoleamine 2,3-dioxygenase (IDO) for the treatment of melanoma through a microneedle-based transcutaneous delivery approach [23].

## 3. The Formulation and Modification of Nanoparticles

The first step for improving nanoparticles is to modify their absorption, distribution, metabolism, and elimination. A pattern emerged as intra-organ nanomaterial delivery because a cell located near the vascular inlet is more likely to take up and accumulate nanomaterial when compared with a cell located near the vascular outlet. The blood clearance mechanism of administered hard nanomaterials is elucidated in relation to blood flow dynamics, organ microarchitecture, and cellular phenotype [24].

Designing considerations, such as size, shape, surface coating, and encapsulation, can be manipulated to prolong blood circulation and enhance treatment efficacy. Nanoparticle size plays a key role in determining the penetration and accumulation in tumor tissue. Small NPs usually have a better effect than large NPs. For smaller nanoparticles, their retention within tumors depends on the frequency of interaction of particles with the perivascular extracellular matrix, whereas transport of larger nanomaterials is dominated by Brownian motion [25]. Shape is another determinant of the particle effect. Discoidal particles tend to accumulate more than spherical particles in most organs except the liver, where cylindrical particles are deposited at a larger extent [26]. Based on the anionic charge of endothelial glycocalyx, it is obvious that cationic NPs extravasate from blood vessels more rapidly than neutral or anionic particles.

Coating particles with an amphiphilic polymer can facilitate water solubility and biocompatibility. Chen et al. compared the biodistribution of iron oxide nanoparticles (IONPs) coated with poly(ethylene oxide)-block-poly(γ-methacryloxypropyltrimethoxysilane) (PEO-b-PyMPS) with that of IONPs coated with either (3-Aminopropyl) trimethoxysilane (ATPM) or dextran only, and consequently found that PEO-b-PyMPS-coated IONPs demonstrated significantly lower distribution to the liver and spleen as compared with dextran-coated or (ATPM)-coated IONPs [27]. Another report showed that dimercaptosuccinic acid-coated magnetite nanoparticles can be used as an efficient drug delivery of IFN-γ, guided by an external magnetic field [28]. It is well established that polyethylene glycol (PEG)-coated nanocarriers reduce immunogenicity, increase the reticuloendothelial system uptake and show high affinity for angiogenic endothelial and tumor cells [29]. Other than synthetic coating materials, the source of biologic coatings is also a good choice. A report showed that cancer cell membrane-coated nanoparticles can promote a tumor-specific immune response for use in vaccine applications, by taking advantage of the membrane functionalization [30].

As for encapsulation, there are many choices, including chemotherapeutic drugs, nucleotide, and protein. DNA-carrying nanoparticles can efficiently introduce leukemia-targeting chimeric antigen receptor genes into T cell nuclei, thereby inducing long-term disease remission [31]. The gold nanocluster (GNC)-assisted delivery of small interfering RNA (siRNA) of nerve growth factor(NGF) (GNC–siRNA) allows efficient NGF gene silencing and pancreatic cancer treatment. The GNC–siRNA complex increases the stability of siRNA, prolongs the circulation lifetime, and enhances the cellular uptake and tumour accumulation [32].

Furthermore, changing the response of nanoparticles can achieve the goal of controlled release. An injectable nanoparticle generator (iNPG) was developed to enhance the delivery of polymeric doxorubicin (pDox), consisting of nanoporous silicon particles packaged with pDox, doxorubicin conjugated to poly by a pH-sensitive cleavable linker [33]. The iNPG-pDox accumulates in tumors as a result of natural tropism, and the p-Dox is transported to the perinuclear region and cleaved into Dox. Besides, the temperature responsive polymer conjugated on the porous silicon nanoparticles provides controlled release by changing its conformation when heated [34].

The engineering of nanoparticles is not only limited to the optimal, physical, and chemical features of the particle itself, but is also determined by a biological system of patients. Researchers use Monte Carlo simulation to model the process of nanoparticle accumulation. They discovered that changes in pathophysiology associated with tumor volume can selectively change tumor uptake of nanoparticles of varying size. This result inspires that nanoparticles can be personalized according to a patient’s disease state for the optimal outcomes [25].

Overall, particle characteristics have a major impact on the biodistribution of nanotechnology-based drugs. Consequently, modification of nanoparticles can optimize the anticancer effect.

### 3.1. Polymeric Nanoparticles

Polymeric nanoparticles are characterized by design flexibility based on functionalization, ploymer diversity, and macromolecular synthesis methods, which are of interest in therapeutic application [35].

The initial polymeric nanoparticles are based on non-biodegradable polymers, such as poly methyl methacrylate, polyacrylamide, polystyrene, and polyacrylates, which cannot be easily degraded and excreted [36]. Biodegradable polymeric particles are increasingly attractive owing to reduced toxicity and increased biocompatibility, including poly(lactide), poly(lactide-co-glycolide) copolymers, and poly(amino acids) [37]. Also, somemsynthetic polymer nanoparticles can be designed to work as a protein affinity reagent. Polymer NPs with nM affinity to a key vascular endothelial growth factor (VEGF165) inhibit binding to its receptor VEGF-2, preventing downstream VEGF165-dependent endothelial migration and invasion into the extracellular matrix, meanwhile, they are not found to exhibit off-target activity [38,39]. Jun et al. reported a multinuclear polymeric nanoparticle, which can load high amounts of proteins and release them in a sustained manner while maintaining bioactivity [40].

### 3.2. Inorganic Nanoparticles

Inorganic nanomaterials have many unique physical properties, such as optical, electrical, magnetic, and thermal properties, and also possess considerably varied nanostructures, such as spheres, tubes, rods or sheets, and so on [41].

The hollow carbon nanospheres (HCN) are constructed as a versatile platform for co-delivery of siRNA targeting multidrug resistance gene (MDR1) mRNA (siMDR1) and chemotherapeutics (Doxorubicin or Cisplatin) with enhanced loading capability to down-regulate more than ~96% of MDR1 protein expression of doxorubicin-resistant breast cancer and leads to ~90% reduction of weight of tumour on mice [42]. Another article demonstrated that ferumoxytol, a kind of iron oxide nanoparticle, inhibits tumor growth by inducing M1-type macrophage polarization in tumor tissues by many in vitro and in vivo studies [43]. Recently, gold nanoparticles have also been explored as the delivery of cancer antigen and immune adjuvant, and are easily accumulated in the immune system and tuned to a desired size or shape. Besides, they can be applied in photothermal ablation and light-triggered drug delivery [44].

### 3.3. Liposomes

The liposomes have soft, deformable, and biodegradable properties. Besides, they are functionally integrated and fulfill the co-delivery of multiple drugs. The disadvantage of liposomes is their poor stability for clinical requirements. Consequently, some engineering approaches are designed to overcome the issues of high cationic charge density and the tendency to form aggregates. For example, some liposomes have been modified with various ligands specific for targeting to particular tissues to enhance the distribution of nucleic acid-bearing liposomes inside target tissues [45]. 

In a study, cationic liposomes loaded with a long synthetic peptide and poly (I/C) showed at least a 25-fold increase over the T cell frequency, showing it to be a promising technique as a powerful therapeutic cancer vaccine formulation [46]. Researchers have developed novel nanogels based on self-assembly of hyaluronic acid-epigallocatechin gallate conjugates, linear polyethylenimine and Granzyme B for the intracellular delivery of the cytotoxic protein Granzyme B for cancer therapy [47].

### 3.4. Exosomes

Exosomes are small biological membrane vesicles that are 30 to 100 nm in diameter, which can be used as pioneering cancer vaccines to enhance the immune system owing to excellent host biodistribution and biocompatibility [48]. There are currently three main classes of exosomes: dendritic cell-derived exosomes, tumor cell-derived exosomes, and ascetic cell exosomes.

A Phase I clinical trial showed that ascetic cell exosomes, combined with a granulocyte-macrophage colony-stimulating factor, could induce a favourable response by producing antitumor cytotoxic T lymphocytes [49]. Also, they could serve as a molecular vehicle for drugs or functional RNA into cells [50].

## 4. The Application of Nanoparticles

### 4.1. Vaccine

A wide range of cancer vaccines has been evaluated for a variety of human malignancies and the goal is to deliver tumor-associated antigens to professional antigen presenting cells to elicit adaptive immune responses mediated by tumor-specific cytotoxic T cells and antibodies. The vaccine in the form of nanoparticles not only antigen stability and immunogenicity, but also targets delivery and control release.

For antigen-negative variant clones that escaped immunosurveillance or underwent immunoediting, Song et al. utilized diverse antigenic determinants from heat shock-treated tumor cells to improve the immunogenicity of DC-based vaccines [51].

Aside from vaccination, co-delivery of tumor antigens and adjuvants is also crucial. Liposome-based particles delivering a model tumor antigen (OVA) in the context of CpG or other toll-like receptor agonists had superior immunogenic activity against melanoma compared with conventional vaccination approaches [52].

### 4.2. Multi-Clone Antibody

Blockage of receptor with antibodies is an important mode of molecular targeted therapy, including the blockage of VEGF, human epidermal growth factor receptor 2 (Her-2), PD-1, and CTLA-4, among others. The biodegradable poly(dl-lactide-co-glycolide) nanoparticle carrying anti-OX40 monoclonal antibody can induce cytoxic lymphocyte proliferation and tumor-specific cytotoxicity [53].

Yuan et al. created a multivalent bi-specific nanobioconjugate engager (mBiNE) to promote selective and enhanced eradication of cancer cells by means of simultaneously targeting HER2 expressed by cancer cells and pro-phagocytosis signaling mediated by calreticulin [54]. The antibody could boost anti-tumor immunity for cancer immunotherapy in alliance with nano-vaccines. The combination of a simple physical mixture of an antigen with a synthetic polymeric nanoparticle, called PC7A nano-vaccine and an anti-PD-1 antibody showed great synergy, with 100% survival over 60 days in a TC-1 tumor model [11].

### 4.3. Delivering of Regulator mRNAs

Apart from antigens and/or antibodies, nanoparticles can also carry messenger RNA, microRNA, long noncoding RNA, and others to the site of tumor cells. The nanoparticle carrier can protect DNA/RNA molecules from degrading during blood circulation, and increase the transfection efficiency into the tumor microenvironment, as well as release with control in tumor site [55]. Utilizing the technique of gene engineering, tumor cells can be killed efficiently by delivering mRNA of tumor suppressor genes or siRNA that can silence the expression of an oncogene. Furthermore, with respect to cancer immunology, the delivered regulator mRNA has the potential to regulate the immune response against tumor cells by the means of targeting some immune mediators.

The RNA-lipoplexes (RNA-LPX) protects RNA from extracellular ribonucleases and enhance targeting to dendritic cell and macrophage populations [56]. Another paper reported that delivering PD-L1 siRNA, with the aid of folic acid (FA)-functionalized polyethylenimine (PEI) polymers can block PD-1/PD-L1 interactions and enhance T cell killing [57].

### 4.4. Artifiicial Antigen Presenting Cells

Antigen presenting cells (APCs), consisting mainly of macrophages and dendritic cells, capture foreign pathogens and display peptides to T cells in the form of antigen-major histocompatibility (MHC)-T cell receptor (TCR), and activate the immune response. Synthetic artificial APCs (aAPCs) are particles that require proteins for T cell activation, such as MHC-epitope or agonist anti-CD28 and agonist anti-CD28, have been conjugated [58]. For example, aAPC composed of poly(lactic-co-glycolic acid) (PLGA) nanoparticles surface-modified with avidin-palmitate conjugates was generated to anchor peptide-MHC and costimulatory ligands to the particle surfaces, which was stimulated to a much greater extent in T-cell activation of in vitro experiments [59].

The first generation aAPCs were composed of solid, micro-sized polystyrene beads or with iron oxide cores and were used for ex vivo expansion of T cells. Compared with first generation, the second generation of aAPCs were smaller particles at the nanometer size scale for in vivo applications, showing favorable distribution to T-cell-rich regions [60].

### 4.5. Others

Recently, artemisinin and its derivatives (ARTs) had shown anticancer activity using in vitro and animal experiments. As the mechanism of Plasmodium, the cell membrane of tumor cells was also the main target of artemisinin attack. The main mechanisms of action of ARTs were the production of reactive oxygen species, inhibition of cell cycle in G_0_/G_1_ phase, induction of apoptosis, and inhibition of angiogenesis [61,62,63].

Natesan S et al. developed artemisinin loaded magnetic nanoparticles, which can be a potential breast cancer drug. Using an external magnetic field, this kind of nanocompound could selectively accumulate the drug at the target site and thereby reduce the doses required to achieve therapeutic concentration, which may otherwise produce serious side effects on healthy cells [64]. Liu R et al. designed novel artesunate-loaded bovine serum albumin nanoparticles to achieve the mitochondrial accumulation of artesunate and induce mitochondrial-mediated apoptosis. They reveal that artesunate-loaded bovine serum albumin nanoparticles damaged the mitochondrial integrity and activated mitochondrial-mediated cell apoptosis by upregulating apoptosis-related proteins and facilitating the rapid release of cytochrome C [65].

## 5. Future Perspective and Challenges

The nanoparticle-based cancer immunotherapy facilitates overcome tumor immune editing responses and obviate the adverse effects caused by systemic inflammation [66]. Because personalized medicine is increasingly becoming the mainstream of cancer therapy, the cross-talk of nanotechnology and immunotherapy in contemporary oncology is full of opportunities and challenges.

Emerging evidence confirms that cancer immunotherapies can generate more robust adaptive and durable antitumor responses in the formulation of nanoparticles. Thus, cancer immunoengineering is a powerful strategy for further consideration and investigation.

With the development of gene therapy and other fields, nanodrugs are no longer confined to traditional chemotherapeutic drugs. They also show great potential in new anticancer drugs such as molecular targeted agents, antisense nucleotides, siRNA, messenger RNA, and DNA oligonucleotide inhibitors, improving gene therapy. Safety also improves the effectiveness of immunotherapy.

Although many tumor nanodrugs are in clinical trials, there is still a long way to go before they are truly adaptive in the field of cancer immunotherapy. To achieve clinical application, nanodrugs need to address at least the following challenges: (1) researching on controllable and renewable nanoparticle synthesis methods; (2) establishing a complete clinical evaluation and monitoring system; (3) implementing good production management practices (GMP); and (4) realizing the transition from basic research to clinical products and commercial production.
